# Patterns of Sleep Quality and Their Associations With Depressive and Anxiety Symptoms Among Chinese Coronary Heart Disease Patients: A Latent Class Analysis

**DOI:** 10.1155/da/2442363

**Published:** 2025-11-14

**Authors:** Shuwen Bai, Wenwen Chen, Qi Li, Jiukai Zhao, Dianjun Qi, Shuang Zang

**Affiliations:** ^1^Department of Public Health and Medicinal Administration, Faculty of Health Sciences, University of Macau, Macao SAR, China; ^2^School of Politics and Public Administration, Wuhan University, Wuhan City, China; ^3^Affiliated Zhongshan Hospital of Dalian University, Dalian City, China; ^4^The Fifth Clinical Medical College, Guangzhou University of Chinese Medicine, Guangzhou City, China; ^5^Department of Community Nursing, School of Nursing, China Medical University, Shenyang City, China; ^6^Department of General Practice, The First Affiliated Hospital of China Medical University, Shenyang City, China

**Keywords:** anxiety symptoms, China, coronary heart disease, depressive symptoms, latent class analysis, sleep quality

## Abstract

**Background:**

Sleep problem among coronary heart disease (CHD) patients has emerged as a pressing health problem. This study aimed to explore different sleep quality patterns and their associations with depressive and anxiety symptoms among CHD patients.

**Methods:**

This study included 691 CHD patients from China and was conducted in 2023. Basic demographic characteristics, sleep quality, depressive, and anxiety symptoms were collected. Latent class analysis (LCA) and binary logistic regression analysis were conducted to identify sleep quality patterns and to explore the associations between these patterns and symptoms of depression and anxiety.

**Results:**

Among the patients, 62.81% reported depressive symptoms and 48.48% had anxiety symptoms. Four sleep quality patterns were identified: “Good sleep group” (55.57%), “Inefficient short sleep group” (14.33%), “Poor sleep group” (8.68%), and “Disturbed sleep group” (21.42%). Compared to the “Good sleep group,” both “Disturbed sleep group” (OR = 4.39, 95% CI: 2.76–6.97) and “Poor sleep group” (OR = 3.92, 95% CI: 2.02–7.61) exhibited high depressive symptoms, with “Inefficient short sleep group” also showing increased depressive symptoms (OR = 2.48, 95% CI: 1.54–4.02). For anxiety symptoms, “Poor sleep group” (OR = 2.90, 95% CI: 1.64–5.12), “Disturbed sleep group” (OR = 2.35, 95% CI: 1.60–3.47), and “Inefficient short sleep group” (OR = 2.12, 95% CI: 1.35–3.31) all exhibited elevated levels of anxiety symptoms. These associations remained robust after adjusting for potential confounders.

**Conclusion:**

This study highlights the critical role of sleep quality in the mental health of CHD patients, identifying specific sleep quality patterns associated with higher risks of depressive and anxiety symptoms. Improving sleep quality may serve as an effective approach to alleviating these mental health symptoms, offering valuable insights for targeted interventions to enhance well-being of CHD patients.

## 1. Introduction

Sleep quality is crucial for human health and is increasingly recognized as a significant public health issue [1], especially among individuals with coronary heart disease (CHD) [2]. Good sleep is essential for cardiovascular health, but the physiological symptoms of CHD, such as chest pain, shortness of breath, and nocturnal dyspnea [3] can directly disrupt patients' sleep. Moreover, hospitalization, the side effects of prolonged medication treatments, and psychological distress related to managing chronic illness, such as anxiety symptoms, further exacerbate sleep problems in CHD patients [4]. Studies indicate that sleep problems are common in CHD patients [5]. These problems, characterized by difficulties in falling asleep, frequent nocturnal awakenings, and nonrestorative sleep [5], increase the risk of further cardiovascular complications.

Beyond physical health impacts, poor sleep quality often leads to psychological distress, such as anxiety and depressive symptoms. Studies have shown a bidirectional relationship between sleep quality and mental health [6]. On the one hand, poor sleep quality can result in increased anxiety and depressive symptoms. On the other hand, individuals suffering from depressive and anxiety symptoms often report greater difficulties falling asleep and maintaining sleep [7]. This bidirectional relationship is especially significant for CHD patients [8]. Poor sleep quality in CHD patients is often associated with increased levels of anxiety and depressive symptoms, which in turn negatively affect sleep quality, creating a vicious cycle [9]. Moreover, this relationship is linked to poorer disease prognosis and higher mortality rates [9]. Research indicates that CHD patients with comorbid depressive or anxiety symptoms are at a higher risk of rehospitalization and death due to the interplay between sleep disturbances and mental health issues [10]. Therefore, studying the association between sleep quality and anxiety symptoms and depressive symptoms in CHD patients is critically important for their overall health and well-being.

While previous studies have focused on the sleep and mental health of CHD patients, these studies have often concentrated on CHD patients who are hospitalized or seeking medical treatment [5]. Such patients are typically in the acute phase of the disease, and the hospital environment itself can influence their sleep quality and mental state [11], limiting the generalizability of these findings to the broader CHD population. Moreover, physiological factors, including chest pain, respiratory disturbances, medication side effects, and compromised cardiac functions have been shown to adversely affect sleep quality in CHD patients [12, 13]. In contrast, this study utilizes a nationwide community-based sampling method to reflect the daily sleep quality and anxiety and depressive symptoms of CHD patients. Thus, this study not only provides more representative results but also reveals the real-world challenges faced by CHD patients in a community setting, offering valuable insights for the development of more effective intervention strategies.

In addition to the focus on specific patient population, previous studies on sleep, depressive, and anxiety symptoms have often concentrated on singular sleep factors, such as sleep duration and interruptions, and their general associations with depressive and anxiety symptoms [5, 14]. Given the complexity of sleep problems and their associations with psychological distress in CHD patients, it is essential to identify specific sleep quality patterns and understand how these patterns relate to mental health outcomes. Latent class analysis (LCA) offers a unique approach which can categorize sleep quality by identifying subgroups within the participants that share similar sleep characteristics. By examining these subgroups, we can better understand the heterogeneity of sleep problems in CHD patients and their distinct relationships with depressive and anxiety symptoms. This study aims to use LCA to explore different sleep quality patterns among CHD patients and investigate how these patterns are associated with depressive and anxiety symptoms, providing insights into the importance of personalized interventions for this vulnerable population.

## 2. Methods

### 2.1. Study Design and Data Collection

This study was conducted from June 20 to August 31, 2023. It involved sampling from the general community population. To ensure randomness in the sampling process, a multi-stage sampling method was used, covering 22 provinces, 5 autonomous regions, 4 direct-controlled municipalities, Hong Kong Special Administration Region, and Macau Special Administration Region. Collectively, these sampled provincial-level administrative regions account for 97.06% of China's administrative coverage [15]. The number of cities sampled was determined based on the population size of each province and autonomous region. By using a random number table, 2–12 cities were randomly selected, resulting in a total of 144 cities. In addition to the four municipalities, the Hong Kong Special Administration Region and the Macau Special Administration Region were directly included, yielding 150 cities in the first sampling stage.

In the second sampling stage, the number of communities that were sampled in the 150 selected cities was based on the population size of each administrative region. Each province sampled 10–60 communities (urban communities/rural villages) from selected cities, with a ratio of three urban communities to two rural villages, totaling 800 communities. Finally, residents in each selected community were quota sampled based on gender and age, with a gender ratio of 1:1 and an age distribution based on the seventh national census [16]. Using disease records from community health service institutions, individuals with confusion, psychiatric abnormalities, or cognitive impairment were excluded. In addition, during questionnaire administration, investigators also asked participants whether they had any mental illness; those who reported mental illness were excluded from the study [17].

The questionnaire survey was distributed via Questionnaire Star, an online survey platform widely used in China. After standardized training, at least one survey team was formed in each city to conduct face-to-face interviews, ensuring survey reliability and accuracy. During these sessions, participants filled in the questionnaires via a link provided on the platform. For participants with cognitive capabilities but mobility challenges, investigators assisted in completing the questionnaire on their behalf without any suggestive words.

After all questionnaires were collected, we screened for participants with CHD. One questionnaire item asked, “Have you ever been diagnosed by a physician with any of the following 13 conditions, including CHD, diabetes, stroke, or others?”. Participants who selected CHD were included in this study. For participants who reported any disease, confirmation was obtained either through records from community health service institutions or through self-report based on a formal diagnosis by a certified hospital.

### 2.2. Study Population

The participants' inclusion criteria were: (1) age ≥ 18 years; (2) having Chinese nationally; (3) residents away from China for less than 1 month; (4) understanding the meaning of each questionnaire item; and (5) selected “CHD” in response to the questionnaire item, “Have you ever been diagnosed by a physician with any of the following 13 conditions?”. The participants' exclusion criteria were: (1) confusion, psychiatric abnormalities, or cognitive impairment; (2) currently participating in other similar studies or having previously participated in PBICR-related surveys; and (3) those who dropped out before the questionnaire completion.

### 2.3. Ethical Considerations

Ethical approval was granted by the Ethics Research Committee of Shandong Provincial Hospital (Number SWYX:2023-198). Informed consents were obtained from all participants. All data were collected anonymously and kept confidential.

### 2.4. Sample Size Calculation

This study used the sample size calculation method for cross-sectional studies to calculate the sample size. The confidence interval was set to 95%, and the margin of error accepted was set to 5%, which means the *α* = 0.05, *Z*_1−*α*/2_ = 1.96. The precision (*d*) was set as 0.05. Based on data from a study [18], we used the expected incidence (*p*) = 0.35. Taking into account an attrition rate of ~20%, the final minimum sample size was 438. Overall, a total of 774 CHD patients were enrolled in this study. The formula is as follows:

  $$\begin{array}{c} N=\;{\left(Z_{1-\alpha /2}\right)}^{2}\times p\times \left(1-p\right)/d^{2}, \end{array}$$

where *N* corresponds to the required sample size; *Z*_1−*α*/2_ is the critical value of the standard normal distribution at the desired confidence level; *p* is the estimated proportion of the population; and *d* is the margin of error or the desired precision.

### 2.5. Measurement

#### 2.5.1. Brief Version of the Pittsburgh Sleep Quality Index (B-PSQI)

The B-PSQI, developed by Buysse et al. [19], was utilized in this study using the Chinese version to assess participants' sleep quality and disturbances over a 1-month period. The Chinese version of B-PSQI has demonstrated good internal consistency and test–retest reliability [20]. It evaluates five domains of sleep: subjective sleep quality, latency, duration, efficiency, and disturbance. Each domain's score ranges from 0 (good sleep or no problems) to 3 (poor sleep or severe problems). The total score ranges from 0 to 15, with higher scores indicate more severe complaints [21]. According to previous studies [22], the score for each domain is categorized into two groups: good (score of <2) and poor (score of ≥2). In this study, the B-PSQI had a Cronbach's alpha value of 0.739.

#### 2.5.2. Patient Health Questionnaire-9 (PHQ-9)

The PHQ-9, developed by Spitzer et al. [23], is a screening tool that assesses participants' depression-related symptoms over the past 2 weeks [24]. The questionnaire consists of 9 items, each scored on a scale from 0 (not at all) to 3 (nearly every day). The total score ranges from 0 to 27, with higher scores indicating more severe depressive symptoms and a score of 5 or higher indicates depressive symptoms [25]. In this study, the PHQ-9 had a Cronbach's α coefficient of 0.911.

#### 2.5.3. 7-Item Generalized Anxiety Disorder (GAD-7)

The 7-item Generalized Anxiety Disorder (GAD-7), developed by Spitzer et al. [26], is a self-report anxiety symptom questionnaire designed to assess the participants' anxiety status during the previous 2 weeks [27]. The GAD-7 included 7 items, each scored on a scale from 0 (not at all) to 3 (nearly every day). The total score ranges from 0 to 21, with higher scores indicating more severe anxiety symptoms and a score of 5 or higher indicates anxiety symptoms [28]. In this study, the GAD-7 had a Cronbach's α coefficient of 0.931.

### 2.6. Covariates

Based on prior studies [29–33], this study identified several potential confounding variables, including sex (male vs. female), ethnicity (Han Chinese vs. Minorities), whether having religion (yes vs. no), age (18–44, 45−64, and ≥65), smoking status (yes vs. no), drinking status (yes vs. no), marital status (married vs. other), BMI (normal vs. abnormal), educational level (high school and below vs. college and above), per capita monthly household income (≤3000 Chinese Yuan, 3001–6000 Chinese Yuan, and ≥6001 Chinese Yuan), place of permanent residence (urban vs. rural), whether family being in debt (yes vs. no), and whether living alone (yes vs. no).

### 2.7. Statistical Analysis

The statistical analysis was performed using SPSS 27 (SPSS Inc., Chicago, IL, USA) and Mplus 7.4 (Muthén & Muthén, Los Angeles, CA, USA). (1) Descriptive statistical analysis was performed to analyze the characteristics of the included population. (2) LCA was used to identify patterns of sleep quality. (3) Chi-square test was used to examine the differences in socio-demographic factors among each latent class. (4) The binary logistic regression was used to explore the association of sleep quality patterns with depressive and anxiety symptom using three models: Model 1 was unadjusted; Model 2 was adjusted for age group and sex; and Model 3 was adjusted for age group, sex, ethnicity, whether having religion, smoking status, drinking status, marital status, BMI, education level, per capita monthly household income, place of permanent residence, solitary living condition, debt situation. The independent variables included “inefficient short sleep group,” “poor sleep group,” “disturbed sleep group,” and “good sleep group,” with sub-variables defined accordingly. The results were considered significant when *p*  < 0.05 (two-sided).

The fitting test indicators of LCA encompassed Akaike Information Criteria (AIC), Entropy, Bayesian Information Criteria (BIC), adjusted BIC (aBIC), Lo-Mendell-Rubin Likelihood Ratio (LMR), and Bootstrapped Likelihood Ratio test (BLRT). Lower values of AIC, BIC, and aBIC were considered better. BIC and aBIC were better indicators of the number of classes than AIC [34]. Entropy index quantified the accuracy of class membership assignment, with higher values signifying higher classification accuracy. The presence of significant BLRT and LMR tests meant that a “K-class-model” performed better than a “K-1-class-model” [35].

### 2.8. Quality Control

Before the investigation began, we consulted experts and conducted three rounds of pre-investigation, incorporating their feedback to refine and improve the questionnaire. All investigators were trained. During the investigation, the investigators summarized, evaluated, and provided feedback on the collected questionnaires each week. After data collection, two researchers performed back-to-back logical checks and data screening to ensure the quality of the questionnaire responses.

## 3. Results

### 3.1. Sample Characteristics

In this study, a total of 774 questionnaires were collected from CHD patients. After identifying and excluding 83 questionnaires due to logical errors, 691 valid questionnaires were retained, resulting in an effective response rate of 89.28%. The prevalences of depressive and anxiety symptoms among CHD patients in this study were 62.81% and 48.48%, respectively.  Table 1 presents the characteristics of socio-demographics of CHD patients in this study, as well as the distribution of these factors among people with different anxiety and depressive symptoms. Of the patients, 83.94% had an education level of high school education and below. Additionally, 72.65% of patients were married, and 30.82% were in family debt.

### 3.2. Identification of Latent Class Models for Sleep Quality

LCA was conducted on the five B-PSQI components to identify different subgroups of sleep quality among CHD patients. On the basis of B-PSQI scores, participants were assigned to one of seven classes (classes 1–7) in line with the model criteria (Table S1). The optimal number of potential categories was determined according to the criteria outlined in the statistical analysis section. As shown in Table S1, only models 2, 3, and 4 exhibited LMR (*P*) and BLRT (*P*) <0.05. Among these three models, model 4 demonstrated lowest BIC and aBIC. After considering the model-fitting index and clinical significance, the four-category model was chosen as the best-fitting model.

Table S2 displays the fitting information for the LCA of different sleep quality subgroups among CHD patients. The average probability of group membership for all three categories exceeds 0.70, suggesting a favorable model fit and reliable results [36].

Table S3 illustrates the likelihood of scoring within each category of sleep problem. This visual representation offers insights into the probability of an individual attaining a specific score in a given sleep problem category.

### 3.3. Identification of Potential Sleep Categories Among CHD Patients

**Figure 1:**
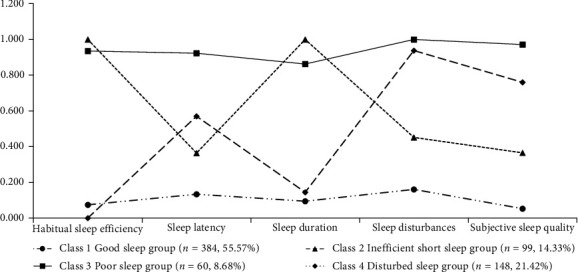
Probability of scoring each sleep problem pattern.

Figure 1 illustrates the distribution of CHD patients across four potential sleep quality patterns. It also displays the respective mean values of the five sleep domains for each category. This visualization provides insight into the relationship between different sleep domains and each pattern.

As shown in Figure 1, class 1 exhibited consistently lower scores across all factors and was consequently labeled as the “Good sleep group” (*n* = 384, 55.57%). Class 2 showed high probabilities of low sleep efficiency and short sleep duration, so it was labeled as “Inefficient short sleep group” (*n* = 99, 14.33%). Class 3 had high probabilities of all B-PSQI components and was labeled as “Poor sleep group” (*n* = 60, 8.68%). Class 4 had relatively high probabilities for sleep onset latency, sleep disturbance, and subjective sleep quality, with sleep disturbance being the highest. Accordingly, we labeled class 4 as “Disturbed sleep group” (*n* = 148, 21.42%).

### 3.4. Distribution Characteristics of Different Types of Sleep Quality Patterns

We further analyzed the distribution of sleep quality patterns across the characteristic of socio-demographics among CHD patients using chi-square test (Table 2). The results showed statistically significantly differences in sleep quality patterns among CHD patients based on age group, marital status, educational level, per capita monthly household income, whether living alone, and whether family being in debt (all *p* value <0.05).

### 3.5. Association Between Sleep Quality Patterns With Depressive and Anxiety Symptom

Table 3 presents the association between different patterns of sleep quality and depressive and anxiety symptoms. In the unadjusted logistic regression analysis, compared to the “good sleep group,” patients in the “disturbed sleep group” (OR = 4.39, 95% CI: 2.76–6.97) and “poor sleep group” (OR = 3.92, 95% CI: 2.02–7.61) exhibited higher levels of depressive symptoms. Additionally, the “inefficient short sleep group” also showed increased depressive symptoms (OR = 2.48, 95% CI: 1.54–4.02). Regarding anxiety symptoms in the unadjusted logistic regression analysis, the “poor sleep group” (OR = 2.90, 95% CI: 1.64–5.12), the “disturbed sleep group” (OR = 2.35, 95% CI: 1.60–3.47), and the “inefficient short sleep group” (OR = 2.12, 95% CI: 1.35–3.31) exhibited increased anxiety symptoms. These association persisted with statistical significance in Model 2, adjusted for sex and age group (*p* < 0.05). In the fully adjusted Model 3, these associations also remained significant, except for anxiety symptoms in the “Inefficient short sleep group.”

## 4. Discussion

To the best of the authors' knowledge, this is the first study to investigate sleep quality patterns among CHD patients in China and to explore the association between these sleep quality patterns and symptoms of depression and anxiety. Furthermore, our study employs a nationwide community-based sampling method, which allows us to capture the real-world sleep quality, anxiety, and depression symptoms of CHD patients in their daily lives, providing more representative results.

In this study, LCA identified four distinct sleep quality patterns among CHD patients. Class 1 (Good sleep group) showed consistently good sleep quality across all dimensions; Class 2 (Inefficient short sleep group) showed inefficient sleep with short duration; Class 3 (Poor sleep group) showed consistently poor sleep quality across all dimensions; Class 4 (Disturbed sleep group) showed prolonged sleep onset, frequently disturbed sleep, and poor subjective sleep quality. Moreover, the prevalence rates of depressive and anxiety symptoms among CHD patients were 62.81% and 48.48%, respectively. The condition of these symptoms varied significantly across different sleep quality patterns. Compared to the “Good sleep group,” both the “poor sleep group” and the “disturbed sleep group” exhibited more depressive and anxiety symptoms. These findings underscore the significant variations in sleep quality among CHD patients and their association with depressive and anxiety symptoms, offering a comprehensive assessment of sleep quality diversity and its association with these mental health issues.

Our results indicated that CHD patients in the “poor sleep group” and “disturbed sleep group” had significantly higher risks of depressive and anxious symptoms compared to those in the “good sleep group.” This finding aligns with previous research on postpartum women, which suggests that poor sleep quality may exacerbate symptoms of depression and anxiety [37]. Firstly, poor sleep quality increases cardiovascular stress [38]. CHD patients already have compromised heart and vascular systems. Inadequate rest and recovery time exacerbate the burden on the heart, worsening disease symptoms. This increased physiological burden further intensifies psychological stress and anxiety. Secondly, poor sleep quality triggers a series of physiological responses [39], such as elevated cortisol levels [40] and activation of the sympathetic nervous system [41]. These physiological changes further deteriorate patients' health and increase anxiety and depressive symptoms [42]. Moreover, CHD patients already face significant psychological stress due to their disease. Poor sleep quality makes it even harder for them to cope with this stress, particularly the risks of sudden cardiac events and fears of mortality, leading to emotional instability and exacerbated depressive and anxious symptoms. Furthermore, good sleep is crucial for physical recovery and repair [43]. Chronic poor sleep in CHD patients results in insufficient rest and repair, causing chronic fatigue and overall deterioration of physical health, which triggers anxiety and depressive symptoms.

Our study also found that CHD patients in both the “poor sleep group” and “disturbed sleep group” experienced frequent sleep disruptions, which were negatively associated with depressive and anxiety symptoms. These findings are consistent with previous research, indicating a negative correlation between sleep disruptions and mood disorders. Frequent sleep interruptions disrupt circadian rhythms [44], which in turn affect the normal expression of clock genes [45], leading to dysregulation of the biological clock and impairing emotional regulation and mental health, particularly in the onset and progression of depressive and anxiety symptoms. A previous study highlights that frequent night awakenings prevent patients from reaching deeper sleep stages [46], which are crucial for emotional regulation and mental recovery [47]. Additionally, the monoamine hypothesis suggests that disruptions in rapid eye movement sleep lead to changes in the levels of monoamine neurotransmitters, which play a crucial role in emotional regulation. Abnormal alterations in these neurotransmitters can result in mood instability and exacerbate depressive symptoms [48]. Sleep disruptions can intensify depressive and anxiety symptoms, further impairing sleep quality and creating a continuous cycle of worsening mental health [6].

Ensuring the sleep quality of CHD patients is critical. Previous studies have demonstrated that sleep education can effectively improve sleep quality and enhance health-related knowledge across various populations [49, 50]. As a preliminary cognitive intervention, sleep education could help patients understand the physiological mechanisms of sleep, its importance for cardiovascular function, and the potential health risks associated with sleep disorders, thereby increasing their motivation and adherence to behavioral change [51]. Following this cognitive activation phase, targeted behavioral interventions are recommended, including sleep hygiene guidance (such as maintaining regular sleep–wake schedules, avoiding stimulants, and optimizing the sleep environment), relaxation training (e.g., diaphragmatic breathing and progressive muscle relaxation), and self-monitoring of sleep behaviors (e.g., through the use of sleep diaries) [52, 53]. This integrated approach, combining cognitive education with behavioral support, is expected to improve both sleep duration and efficiency, promote better overall health and psychological well-being, and reduce unnecessary healthcare utilization—particularly among high-risk CHD patients characterized by short sleep duration and low sleep efficiency [54, 55].

Appropriate levels of physical activity have been shown to contribute positively to the rehabilitation of CHD patients, supporting both cardiovascular function and overall health [56]. Given that poor sleep is common among CHD patients [5] and is associated with worse health outcomes [2], incorporating exercise-based interventions into sleep management strategies may offer dual benefits, enhancing both sleep quality and cardiac recovery. In addition to physical activity, light therapy has demonstrated potential in improving sleep among CHD patients. A randomized controlled trial in older adults with CHD reported significant improvements in sleep quality following light therapy [57], suggesting that light therapy may be valuable as an adjunctive intervention. Moreover, cognitive behavioral therapy has been shown to effectively treat sleep disturbances in individuals with CHD [58]. Providing tailored sleep enhancement strategies, especially for vulnerable subgroups, may not only improve sleep quality but also help alleviate comorbid symptoms of depression and anxiety.

There are still some limitations in this study. Firstly, as a cross-sectional study, it cannot establish causal relationships between sleep quality, depressive symptoms, and anxiety symptoms among CHD patients. Secondly, all variables in this study were based on self-reports, which may introduce self-report bias and common-methods variance. Thirdly, the assessment of anxiety and depressive symptoms relied on self-reported questionnaires. The results might differ if these symptoms had been evaluated through clinical assessment and diagnosis rather than screening tools. Fourthly, we collected only basic demographic, psychological, and behavioral information for CHD patients and did not obtain detailed data on disease-specific physiological symptoms or clinical characteristics, which may influence sleep quality as well as anxiety and depressive symptoms. Fifthly, the sampling was based on the gender distribution of the general population rather than that of CHD patients, which may have affected the representativeness of the CHD sample, particularly in gender-related analyses. Sixthly, this study did not investigate whether general participants had recently used anxiolytics, antidepressants, or hypnotics, which could have affected the results. Seventhly, a relatively high proportion of participants had lower levels of education. While this reflects the current reality among older adults in China, it may have hindered their comprehension and affected the accuracy of their responses. Finally, the CHD patients were selected from the general population. Although the sample size is sufficient for this study, it may not fully represent all CHD patients in China.

## 5. Conclusion

In conclusion, this study highlights the significant association between sleep quality and the mental health of CHD patients, identifying distinct sleep quality patterns associated with the increased risks of depressive and anxiety symptoms. These findings underscore the critical role of sleep quality in CHD management, emphasizing its potential to alleviate symptoms of anxiety and depression in this population. This research provides valuable insights for caregivers, healthcare institutions, and individuals, helping them address the sleep quality issues of CHD patients with different sleep characteristics, thereby reducing their anxiety and depressive symptoms.

## Figures and Tables

**Table 1:** Prevalence of depressive and anxiety symptoms by socio-demographics.

Variable	Total (*n* = 691)		Depressive symptom		Anxiety symptom	
	*n*	Constituent ratio (%)	*n*	Prevalence rate (%)	*n*	Prevalence rate (%)
Sex						
Male	353	51.09	223	63.17	174	49.29
Female	338	48.91	211	62.43	161	47.63
Ethnicity						
Han Chinese	600	86.83	366	61.00	277	46.17
Minorities	91	13.17	68	74.73	58	63.74
Whether having religion						
No	592	85.67	371	62.67	281	47.47
Yes	99	14.33	63	63.64	54	54.55
Age group (years)						
18–44	58	8.39	44	75.86	38	65.52
45–64	248	35.89	165	66.53	122	49.19
≥65	385	55.72	225	58.44	175	45.45
Smoking status						
No	534	77.28	332	62.17	247	46.25
Yes	157	22.72	102	64.97	88	56.05
Drinking status						
No	559	80.90	350	62.61	266	47.58
Yes	132	19.10	84	63.64	69	52.27
Marital status						
Other	189	27.35	134	70.90	105	55.56
Married	502	72.65	300	59.76	230	45.82
BMI						
Normal	396	57.31	242	61.11	184	46.46
Abnormal	295	42.69	192	65.08	151	51.19
Educational level						
High school and below	580	83.94	351	60.52	270	46.55
College and above	111	16.06	83	74.77	65	58.56
Per capita monthly household income (CNY)						
≤3000	264	38.21	168	63.64	134	50.76
3001–6000	314	45.44	191	60.83	145	46.18
≥6001	113	16.35	75	66.37	56	49.56
Place of permanent residence						
Rural	313	45.30	205	65.50	148	47.28
Urban	378	54.70	229	60.58	187	49.47
Solitary living condition						
No	556	80.46	329	59.17	249	44.78
Yes	135	19.54	105	77.78	86	63.70
Debt situation						
No	478	69.18	269	56.28	200	41.84
Yes	213	30.82	165	77.46	135	63.38

Abbreviation: CNY, Chinese Yuan.

**Table 2:** Distribution of sociodemographic characteristics in sleep quality patterns (*n* = 691).

Variable	Class 1 Good sleep group (*n* (%))	Class 2 Inefficient short sleep group (*n* (%))	Class 3 Poor sleep group (*n* (%))	Class 4 Disturbed sleep group (*n* (%))	*p*
Gender					0.905
Male	194 (54.96)	54 (15.30)	30 (8.50)	75 (21.25)	
Female	190 (56.21)	45 (13.31)	30 (8.88)	73 (21.60)	
Ethnicity					0.840
Han Chinese	337 (56.17)	86 (14.33)	51 (8.50)	126 (21.00)	
Minorities	47 (51.65)	13 (14.29)	9 (9.89)	22 (24.18)	
Whether having religion					0.576
No	326 (55.07)	85 (14.36)	55 (9.29)	126 (21.28)	
Yes	58 (58.59)	14 (14.14)	5 (5.05)	22 (22.22)	
Age group (years)					**<0.001**
18–44	29 (50.00)	18 (31.03)	2 (3.45)	9 (15.52)	
45–64	163 (65.73)	25 (10.08)	13 (5.24)	47 (18.95)	
≥65	192 (49.87)	56 (14.55)	45 (11.69)	92 (23.90)	
Smoking status					0.165
No	302 (56.55)	68 (12.73)	46 (8.61)	118 (22.10)	
Yes	82 (52.23)	31 (19.75)	14 (8.92)	30 (19.11)	
Drinking status					0.555
No	313 (55.99)	75 (13.42)	50 (8.94)	121 (21.65)	
Yes	71 (53.79)	24 (18.18)	10 (7.58)	27 (20.45)	
Marital status					**<0.001**
Married	306 (60.96)	56 (11.16)	41 (8.17)	99 (19.72)	
Other	78 (41.27)	43 (22.75)	19 (10.05)	49 (25.93)	
BMI					0.455
Normal	229 (57.83)	51 (12.88)	32 (8.08)	84 (21.21)	
Abnormal	155 (52.54)	48 (16.27)	28 (9.49)	64 (21.69)	
Educational level					**<0.001**
High school and below	331 (57.07)	68 (11.72)	49 (8.45)	132 (22.76)	
College and above	53 (47.75)	31 (27.93)	11 (9.91)	16 (14.41)	
Per capita monthly household income (CNY)					**<0.001**
≤3000	134 (50.76)	47 (17.80)	16 (6.06)	67 (25.38)	
3001–6000	192 (61.15)	28 (8.92)	29 (9.24)	65 (20.70)	
≥6001	58 (51.33)	24 (21.24)	15 (13.27)	16 (14.16)	
Place of permanent residence					0.067
Rural	188 (60.06)	37 (11.82)	21 (6.71)	67 (21.41)	
Urban	196 (51.85)	62 (16.40)	39 (10.32)	81 (21.43)	
Whether living alone					**0.021**
No	323 (58.09)	71 (12.77)	49 (8.81)	113 (20.32)	
Yes	61 (45.19)	28 (20.74)	11 (8.15)	35 (25.93)	
Whether family being in debt					**0.002**
No	286 (59.83)	55 (11.51)	39 (8.16)	98 (20.50)	
Yes	98 (46.01)	44 (20.66)	21 (9.86)	50 (23.47)	

*Note:* As a result of our rounding of decimal points, the sum of percentage of some items may not be 100.00%. Bolded values are significant at *p* < 0.05.

Abbreviation: CNY, Chinese Yuan.

**Table 3:** Association between latent class of sleep quality with depressive and anxiety symptoms.

Model	Variable	Depressive symptoms		Anxiety symptoms	
		OR (95% CI)	*p*-Value	OR (95% CI)	*p*-Value
Model 1	Constant	1.02	0.838	0.64	<0.001
	Inefficient short sleep group	2.48 (1.54–4.02)	**<0.001**	2.12 (1.35–3.31)	**0.001**
	Poor sleep group	3.92 (2.02–7.61)	**<0.001**	2.90 (1.64–5.12)	**<0.001**
	Disturbed sleep group	4.39 (2.76–6.97)	**<0.001**	2.35 (1.60–3.47)	**<0.001**
	Good sleep group	1.00		1.00	

Model 2	Constant	1.96	0.049	1.29	0.410
	Inefficient short sleep group	2.57 (1.57–4.21)	**<0.001**	2.06 (1.30–3.26)	**0.002**
	Poor sleep group	4.74 (2.41–9.31)	**<0.001**	3.25 (1.82–5.81)	**<0.001**
	Disturbed sleep group	4.91 (3.06–7.87)	**<0.001**	2.50 (1.69–3.70)	**<0.001**
	Good sleep group	1.00		1.00	

Model 3	Constant	2.50	0.084	1.40	0.478
	Inefficient short sleep group	2.14 (1.25–3.66)	**0.005**	1.63 (0.99–2.66)	0.054
	Poor sleep group	4.38 (2.17–8.81)	**<0.001**	2.93 (1.61–5.34)	**<0.001**
	Disturbed sleep group	4.79 (2.93–7.82)	**<0.001**	2.25 (1.49–3.38)	**<0.001**
	Good sleep group	1.00		1.00	

*Note:* Model 1 was unadjusted. Model 2 was adjusted for age group and sex. Model 3 was adjusted for age group, sex, ethnicity, whether having religion, smoking status, drinking status, marital status, BMI, education level, per capita monthly household income, place of permanent residence, solitary living condition, debt situation. Bolded values are significant at *p* < 0.05.

## Data Availability

The data that support the findings of this study are available from the corresponding author upon reasonable request.

## Supporting Information

Supporting Information Table S1. The fitting information of sleep latent class model for coronary heart disease patients. Table S2. Average probability of attribution (column) for subjects (rows) in each potential category. Table S3. Probability of scoring each sleep problem category.
