# Supplementary material for: Patterns of Sleep Quality and Their Associations With Depressive and Anxiety Symptoms Among Chinese Coronary Heart Disease Patients: A Latent Class Analysis
Source: Depress Anxiety. 2025 Nov 14;2025:2442363. doi: 10.1155/da/2442363 (PMC12638143; doi:10.1155/da/2442363)
Supplement: Supporting Information — Table S1. The fitting information of sleep latent class model for coronary heart disease patients. Table S2. Average probability of attribution (column) for subjects (rows) in each potential category. Table S3. Probability of scoring each sleep problem category. [file 2442363.f1.docx]

**Supplementary materials**

Supplementary Table 1. The fitting information of sleep latent class model for coronary heart disease patients

Supplementary Table 2. Average probability of attribution (column) for subjects (rows) in each potential category

Supplementary Table 3. Probability of scoring each sleep problem category

Table S1. The fitting information of sleep latent class model for coronary heart disease patients

| Number of classes | AIC | BIC | SSA-BIC | Entropy | LMRT | BLRT | Smallest class (%) |
| --- | --- | --- | --- | --- | --- | --- | --- |
| 1 | 4374.822 | 4397.513 | 4381.637 | - | - | - | 100.000 |
| 2 | 3929.901 | 3979.820 | 3944.894 | 0.710 | <0.0001 | <0.0001 | 42.692 |
| 3 | 3716.838 | 3793.987 | 3740.009 | 0.858 | <0.0001 | <0.0001 | 21.852 |
| 4 | 3681.456 | 3785.833 | 3712.804 | 0.853 | 0.0001 | <0.0001 | 8.683 |
| 5 | 3688.811 | 3820.417 | 3728.338 | 0.899 | 0.1785 | 0.6667 | 8.683 |
| 6 | 3698.690 | 3857.525 | 3746.394 | 0.882 | 0.4588 | 1.0000 | 0.000 |
| 7 | 3709.697 | 3895.761 | 3765.579 | 0.707 | 1.0000 | 1.0000 | 2.315 |

Footnotes:

AIC: Akaike Information Criterion. BIC: Bayesian Information Criterion. SSA-BIC: Simple-Size Adjusted BIC. LMRT: Lo-Mendell-Rubin Test. BLRT: Bootstrapped Likelihood Ratio Test.

Class proportions indicate the proportion of each class in each classification model.

Table S2. Average probability of attribution (column) for subjects (rows) in each potential category

| Class | Class 1 | Class 2 | Class 3 | Class 4 |
| --- | --- | --- | --- | --- |
| Class 1 | 0.950 | 0.000 | 0.001 | 0.049 |
| Class 2 | 0.027 | 0.913 | 0.060 | 0.000 |
| Class 3 | 0.001 | 0.095 | 0.904 | 0.000 |
| Class 4 | 0.086 | 0.000 | 0.028 | 0.886 |

Table S3. Probability of scoring each sleep problem category

| Brief Version of the Pittsburgh Sleep Quality Index | Class 1 Good sleep group (n=384, 55.57%) | Class 2 Inefficient short sleep group (n=99, 14.33%) | Class 3 Poor sleep group (n=60, 8.68%) | Class 4 Disturbed sleep group (n=148, 21.42%) |
| --- | --- | --- | --- | --- |
| Habitual sleep efficiency | 0.075 | 1.000 | 0.935 | 0.000 |
| Sleep latency | 0.134 | 0.364 | 0.923 | 0.570 |
| Sleep duration | 0.095 | 1.000 | 0.863 | 0.145 |
| Sleep disturbances | 0.161 | 0.451 | 1.000 | 0.938 |
| Subjective sleep quality | 0.053 | 0.365 | 0.971 | 0.760 |
